# Fecal Hemoglobin Concentration, a Good Predictor of Risk of Advanced Colorectal Neoplasia in Symptomatic and Asymptomatic Patients

**DOI:** 10.3389/fmed.2019.00091

**Published:** 2019-05-03

**Authors:** Mercedes Navarro, Gonzalo Hijos, Teresa Ramirez, Ignacio Omella, Patricia Carrera-Lasfuentes, Ángel Lanas

**Affiliations:** ^1^Service of Digestive Diseases, University Clinic Hospital, Zaragoza, Spain; ^2^Service of Pathology, University Clinic Hospital, Zaragoza, Spain; ^3^University of Zaragoza, Zaragoza, Spain; ^4^CIBERehd, Madrid, Spain; ^5^IIS Aragón, Zaragoza, Spain

**Keywords:** colorectal adenocarcinoma, fecal occult blood detection, adenoma, symptom, screening

## Abstract

**Background:** Periodical fecal immunochemical testing (FIT) is a cost-effective strategy in colon cancer screening programmes. FIT is also used as a diagnostic test in symptomatic patients, but data, are scarce.

**Aim:** To determine the association between FIT-Hb concentration and the risk of advanced neoplasia (AN) detected in colonoscopy in two different populations.

**Methods:** The outcomes of colonoscopies performed after a positive FIT (>117 ng/ml) (Sentinel Gold test) result were analyzed in patients included within a population-based CRC screening programme (screening group) and, as diagnostic evaluation in symptomatic patients (symptomatic group). The study was performed between January 1st, 2014 and October 31, 2016. Data are reported as medians with interquartile ranges or frequencies and percentages. Positive predictive value (PPV) at arbitrary fecal hemoglobin concentrations were also reported calculated for AN.

**Results:** We recruited 2742 patients who underwent a colonoscopy procedure, 1515 (53.5%) of them within the CRC screening programme. Patients in the screening group were younger (65.0 ± 3.3 vs. 66.2 ± 13.4 years, *p* < 0.001) and more frequently male (*p* < 0.001) vs. the symptomatic group. Colonoscopy found more frequently neoplastic lesions in the screening compared to the symptomatic group (61.9 vs. 44.8% *p* < 0.001). Hb concentration in FIT was significantly higher in patients with AN compared with patients without AN in both groups (*p* < 0.001). The age-adjusted risk of AN increased significantly in both groups according to FIT Hb concentration in the Quartile 3 [OR (95% CI): 2.94 (2.33–3.71)] and Quartile 4 [OR: 5.52 (4.36–6.99)]. Males, in both groups showed a higher probability of presenting AN. FIT values were higher for left- than for right-sided AN in the screening, but not in the symptomatic group. Positive predictive values for AN were higher in the screening group in positive FIT tests (range 43.9–70.5%; 117 to >1,000 ng/ml) compared to those in the symptomatic group (36.3–52.5%). Similar trends were observed for cancer diagnosis alone.

**Conclusions:** Male gender, age, and FIT Hb concentration are predictors of risk of advanced adenoma and colorectal cancer and can be used to prioritize colonoscopy in patients with suspected advanced neoplasia, both in screening and in symptomatic patients.

## Background

Colorectal cancer (CRC) is one of the most commonly diagnosed cancers worldwide, being ranked in prevalence as the third in men and second in women. There are large variations in its incidence and mortality among regions (1). As screening appears to be cost-effective compared to non-screening (2–4), population-based screening programmes have been implemented around the world in the past years (5, 6). Between them, the most common test used as a screening tool in organized screening programmes was the fecal occult blood test, being the fecal immunochemical test (FIT) the most commonly used (6). On the other hand, as FIT is a user-friendly test, that only requires a single sample, without prior dietary restrictions needed (7), is being more frequently used in clinical practice as a diagnostic test for evaluation of patients that refer gastrointestinal symptoms such as change in bowel habits, diarrhea, abdominal pain or anemia prior to colonoscopy (8–10).

Lately, due to the increase in the participation in screening programmes and the sensitivity of the test compared to the guaiac based fecal occult blood test previously used, there has been an increase in the demand for colonoscopies, which has resulted in longer waiting times for patients. Prioritization of patients with a higher risk for presenting an advanced colorectal neoplasia (AN) based on analytic or clinical parameters could mitigate a potential negative impact on waiting lists and on patients' prognosis.

As FIT is a quantitative test, a cut-off value can be chosen to adapt each local programme to the availability of endoscopic resources (11, 12). Recent studies suggest fecal hemoglobin concentration detected in the test can be a predictor of risk of advanced colorectal neoplasia in screening programmes (13–20) and could be used with other variables to stratify the risk of patients prior to colonoscopy in patients with symptoms, but data is still scarce (21–23) and no studies have compared both strategies in the same area of influence. In this study we seek to determine the association between FIT Hb concentration and the risk and positive predictive values of advanced neoplasia detected in colonoscopy in two different populations, symptomatic patients and people undergoing colonoscopy within a population–based CRC screening programme.

## Methods

### Study Population

This retrospective observational study consisted of patients referred to a general tertiary hospital between 1 January 2014 and 31 October 2016 for colonoscopy after a positive FIT performed in two different scenarios:

- Screening group: asymptomatic patients aged 60–69 years old included within a population-based CRC screening programme who tested positive for FIT.
- Symptomatic group: patients referred for colonoscopy due to gastrointestinal symptoms (e.g., alterations of bowel habits, constipation, anemia, diarrhea, etc.) who also tested positive for FIT as a diagnostic evaluation prior to colonoscopy.

Exclusion criteria in the screening group were as follows: personal history of CRC, adenoma or inflammatory bowel disease, familiar history of hereditary CRC, or severe co-existing illness. There were no exclusion criteria in the symptomatic group if tested positive for FIT. FIT negative patients were not included in the study.

### Fecal Immunochemical Test

Patients were instructed how to collect a fecal sample according to the written instructions given with the commercial kit, which included no dietary or medication restrictions. The fecal material was collected in a sampling tube and analyzed using FOB-GOLD® (Sentifit; Sysmex-Sentinel Ch SpA, Barcelona, Spain). The cut-off value applied was 117 ng/ml of buffer (equivalent to 20 micrograms of Hb per gram of feces).

### Colonoscopy, Histologic Examination, and Definitions

Colonoscopies were performed by experienced gastroenterologists of the Service of Digestive Diseases of our center. Polypoid lesions detected in the procedure were removed and classified according to the Spanish Network of Cancer Screening Programs (Red de Programas de Cribado de Cancer; http://www.cribadocancer.es/) which was based on the European guidelines for quality assurance in colorectal cancer screening and diagnosis (24) by an experienced pathologist. Classification included “Low-risk adenomas” defined as 1–2 tubular adenomas <1 cm with low grade dysplasia; “Intermediate-risk adenomas” defined as ≥3 adenomas, or those ≥ 1cm, villous histology or high grade dysplasia; and “High-risk adenomas” defined as ≥10 adenomas or those ≥2 cm. Advanced neoplasia was defined by the European Society of Gastrointestinal Endoscopy (ESGE) (25) as the presence of colorectal cancer or colorectal adenoma with villous histology or high grade dysplasia or >10 mm in size, which includes both the intermediate- and high-risk adenomas defined above. Tumor staging was established according to TNM classification system of the Union for International Cancer Control (26). In this study we have considered right-sided lesions included those found in the cecum, ascending colon, hepatic flexure and proximal transverse colon. Left-sided lesions included those found in the sigmoid, descending colon, splenic flexure and distal transverse colon. Rectal lesions were identified in a different group, but reported together as left-sided colorectal lesions.

### Endpoint of the Study

The primary endpoint was to establish the association between the hemoglobin concentration detected in the fecal immunochemical test and the risk of advanced neoplasia, as defined above by the ESGE, found in the colonoscopy in the two different populations. Secondary endpoints were:

- To evaluate the risk of colorectal cancer according to FIT concentrations.
- To evaluate the positive predictive value of different cut-off values of FIT for cancer and cancer + high-risk + intermediate-risk adenoma, globally and in each group.
- To identify additional independent risk factors for advanced neoplasia.

### Statistical Analysis

Continuous variables were reported as mean with standard deviation (SD) or median with interquartile range (IQR), whereas qualitative variables were expressed as frequencies and percentages. The relationship between qualitative variables was analyzed by contingency tables with Chi-square test. The Kruskal–Wallis test was performed to evaluate differences in fecal hemoglobin concentrations among groups of individuals with different colonoscopy findings. The Mann–Whitney *U* test was used to compare differences between two independent groups. The positive predictive value (PPV) at arbitrary fecal hemoglobin concentrations was calculated for advanced colorectal neoplasia. A logistic regression analysis was performed to determine the independent association of sex, age and FIT quartiles with the detection of AN; ORs (CI95%) were reported. For all tests, a two-sided *p* < 0.05 was considered statistically significant. The statistical analysis was performed using the SPSS software v 22.0 for Windows (SPSS Ibérica, Madrid, Spain).

## Results

A total of 2,742 patients were included in the study, 1,515 in the CRC screening group (55.3%), and 1,227 (44.7%) in the symptomatic group. More men than women participated in the study (57.8% men; 1,585). The mean age of patients was 65.6 ± 9.3 years old, with the youngest being 18 and the eldest 100 years old. In the screening group, patients were younger (65.0 ± 3.3 vs. 66.2 ± 13.4 years, *p* < 0.001) and more frequently male (61.5 vs. 53.3%, *p* < 0.001) compared to the symptomatic group (Table 1). Neoplastic lesions were found in colonoscopies more frequently in the screening group than in the symptomatic group (61.9 vs. 44.8%, *p* < 0.001) (Table 1).

**Table 1:** Demographics and colonoscopy findings according to FIT indication.

	**Global *n* = 2,742**	**Screening group *n* = 1,515**	**Symptomatic group** ***n* = 1,227**	***p*-value**
Sex (men)	1585 (57.8%)	931 (61.5%)	654 (53.3%)	**<0.001**
Age (Mean ±*SD*)	65.6 ± 9.3	65.0 ± 3.3	66.2 ± 13.4	**<0.001**
Colonoscopy findings				**<0.001**
Normal	1254 (45.7%)	577 (38.1%)	677 (55.2%)	
Low-risk adenoma	384 (14.0%)	273 (18.0%)	111 (9.0%)	
Intermediate-risk aadenomaadenoma	630 (23.0%)	408 (26.9%)	222 (18.1%)	
High-risk adenoma	267 (9.7%)	191 (12.6%)	76 (6.2%)	
Cancer	207 (7.5%)	66 (4.4%)	141 (11.5%)	

*Bold values highlight the statistically significant data*.

### Fecal Hemoglobin Concentration According to Colonoscopy Findings

There were statistically significant differences between Hb concentrations in FIT and endoscopic findings, both among the different lesions within each group (the higher the severity of the lesion, the higher the FIT value) and between groups. Overall, hemoglobin FIT values were higher in the symptomatic group (*p* < 0.05), except for high-risk adenomas (Table 2).

**Table 2:** Hemoglobin FIT values according to colonoscopy findings.

**Colonoscopy findings**	**Screening group *n* = 1,515**	**Symptomatic group *n* = 1,227**	***p*-value^**a**^**
Normal	275.0 (169.5 – 572.0)	386.0 (189.5 – 1276.0)	**<0.001**
Low-risk adenoma	264.0 (167.0 – 582.0)	356.0 (180.0 – 834.0)	**0.038**
Intermediate-risk adenoma	499.0 (230.0 – 1245.0)	674.5 (319.8–2837.0)	**0.003**
High-risk adenoma	1249.0 (515.0 – 5429.0)	1797.5 (384.3 – 6159.5)	0.996
Cancer	3604.5 (578.8 – 9451.8)	5845.0 (767.0 – 13967.0)	**0.035**
*p*-value^b^	**<0.001**	**<0.001**	

*Median (Q1–Q3). Bold values highlight the statistically significant data*.

a *Comparison between “SCREENING GROUP” and “SYMPTOMATIC GROUP” groups*.

b *Comparison between colonoscopy findings within each group*.

Colonoscopy showed that 40.3% of the population of the study had AN, 35.8% in symptomatic group (27.1% left-sided, 8.7% right-sided), and 43.9% in the population-based screening programme (23.4% left-sided, 18.8% right-sided) (*p* < 0.001). Fecal hemoglobin values were statistically different between those patients who had or did not have AN or cancer alone, in each group (Table 3).

**Table 3:** Hemoglobin FIT values according to advanced neoplasia and colorectal cancer alone.

	**Global *n* = 2,742**	**Screening group *n* = 1,515**	**Symptomatic group** ***n* = 1,227**	***p*-value^**a**^**
**ADVANCED NEOPLASIA** **(ADVANCED ADENOMA + CANCER)**				
Yes	765.0 (302.5–4543.0)	717.0 (271.5–3841.0)	1065.0 (394.0–5993.0)	**<0.001**
No	305.5 (178.8–740.0)	272.5 (169.0–572.3)	379.0 (189.0–1149.5)	**<0.001**
*p*-value^b^	**<0.001**	**<0.001**	**<0.001**	
**COLORECTAL CANCER**				
Yes	4906.0 (719.0–12699.0)	3604.5 (578.8–9451.8)	5845.0 (767.0–13967.0)	**0.035**
No	394.0 (200.0–834.0)	353.0 (194.0–770.0)	439.0 (211.5–2101.0)	**<0.001**
*p*-value^b^	**<0.001**	**<0.001**	**<0.001**	

*Median (Q1–Q3). Bold values highlight the statistically significant data*.

a *Comparison between “SCREENING GROUP” and “SYMPTOMATIC GROUP” groups*.

b *Comparison between colonoscopy findings within each group*.

### Risk Stratification for Advanced Colorectal Neoplasia

With regard to quartile values, patients were classified in four groups, according to their fecal hemoglobin concentration in FIT, and the risk of advanced neoplasia, considering Q1 as the reference group. As shown in Table 4A, the risk of AN was higher as the fecal hemoglobin concentration increased, globally and in each group separately. A similar pattern was observed when colorectal cancer was considered alone as an outcome, although statistically significance was not reached for Q3 in the screening group and both ranges and ORs were a bit higher in the symptomatic group (Table 4B).

**Table 4A:** Risk of advanced neoplasia (advanced adenoma + cancer) according to Hb quartil.

		**Global *n* = 2,742**	**Range**	**Screening group *n* = 1,515**	**Range**	**Symptomatic group** ***n* = 1,227**
Q_1_	<270	1	<196	1	<223	1
Q_2_	270–430	**1.42 (1.11 – 1.81)**	196 – 370	1.27 (0.93 – 1.74)	223 – 513	**1.65 (1.12 – 2.42)**
Q_3_	431–1,956	**2.94 (2.33 – 3.71)**	371 – 769	**2.58 (1.89 – 3.52)**	514 −3321 3321	**3.14 (2.17 – 4.53)**
Q_4_	≥1,957	**5.52 (4.36 – 6.99)**	≥770	**5.80 (4.26 – 7.90)**	≥3322	**5.55 (3.84 – 8.01)**

*Bold values highlight the statistically significant data*.

**Table 4B:** Risk of colorectal cancer according to Hb quartil.

		**Global *n* = 2,742**	**Range**	**Screening group *n* = 1,515**	**Range**	**Symptomatic group** ***n* = 1,227**
Q_1_	<270	1	<196	1	<223	1
Q_2_	270–430	0.68 (0.33 – 1.38)	196 – 370	0.70 (0.22 – 2.21)	223 – 513	0.75 (0.32 – 1.73)
Q_3_	431–1,956	**2.50 (1.45 – 4.32)**	371 – 769	2.05 (0.81 – 5.21)	514 – 3321	**2.50 (1.28 – 4.87)**
Q_4_	≥1,957	**8.11 (4.94 – 13.30)**	≥770	**5.76 (2.55 – 13.01)**	≥3322	**8.82 (4.80 – 16.21)**

*OR (CI 95%). Bold values highlight the statistically significant data*.

**Figure 1:**
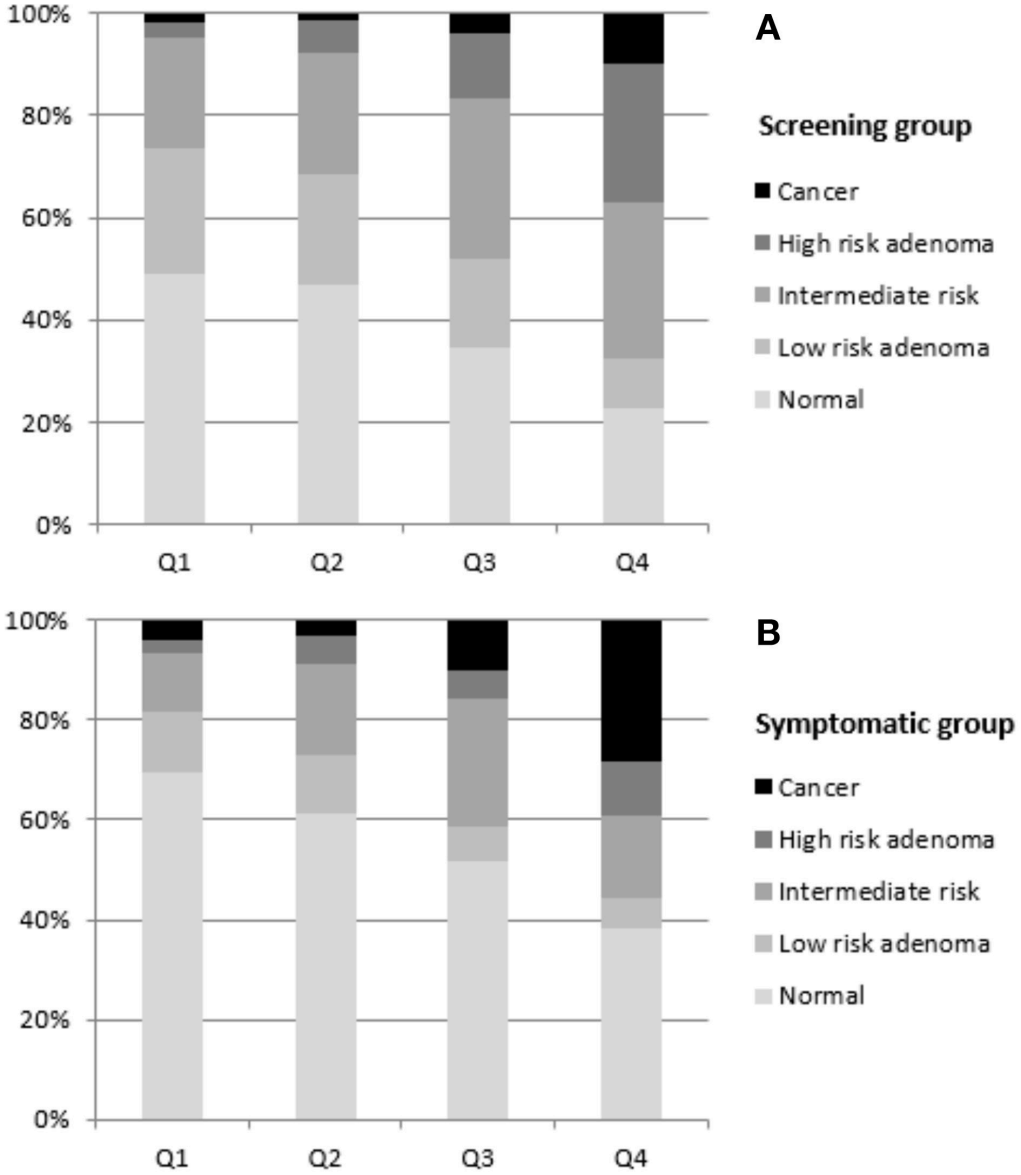
Colonoscopy findings according to Hb quartile in **(A)** screening group (*p* < 0.001) and in **(B)** symptomatic group (*p* < 0.001).

Findings of colonoscopies were also different according to quartiles of the fecal hemoglobin concentration, globally and in each group. The proportion of patients with cancer or a high-risk adenoma increased progressively with each quartile from Q1 to Q4 (Figure 1).

### Effect of Age and Sex on the Risk of Advanced Neoplasia

The mean age of patients was significantly different according to the colonoscopy findings in the symptomatic group (*p* < 0.001), but not in the screening group (*p* = 0.075), probably due to the characteristics of the study population invited to the programme which was between 60 and 69 years old (data not shown**)**. More severe endoscopic findings were observed in elder patients. In the symptomatic group, the adjusted-risk of presenting advanced neoplasia increased 1.01 (CI95%; 1.009–1.02) times per each additional year.

Patients older than 60 years old in the symptomatic group had 1.84 (CI 95%; 1.39–2.44) times more risk of presenting an advanced neoplasia than younger ones, whereas in the screening group the risk was 1.04 (CI95%; 0.52–1.94).

Sex was also found to be an independent risk factor of presenting advanced neoplasia, both globally and in each group (*p* < 0.001). The proportion of men increases with the severity of endoscopic lesion (data not shown). Globally, men presented 2.72 (CI95% 2.31–3.20) times more risk of presenting an advanced neoplasia than women. Similar findings were obtained when each group was analyzed separately, 2.66 (CI95% 2.13–3.31) in the screening group, and 2.68 (2.10–3.34) in the symptomatic group. In the multivariate analysis, the risk of presenting an advanced neoplasia was higher in the male group and in patients with the highest values of hemoglobin concentration in the FIT (Table 5A). Similar trends can be observed when cancer was analyzed as a single outcome (Table 5B), but risk differences are stronger for both men and women in the symptomatic group.

**Table 5A:** Risk of advanced neoplasia according to sex and hemoglobin quartile.

	**Screening group** ***n*** **= 1,515**		**Symptomatic group** ***n*** **= 1,227**	
	**Women**	**Men**	**Women**	**Men**
Q1	1	**2.34 (1.43 – 3.84)**	1	**2.88 (1.55 – 5.34)**
Q2	1.14 (0.66 – 1.96)	**3.40 (2.08 – 5.55)**	1.22 (0.62 – 2.43)	**4.91 (2.74 – 8.81)**
Q3	**2.85 (1.65 – 4.94)**	**5.37 (3.33 – 8.67)**	**3.17 (1.73 – 5.80)**	**9.41 (5.23 – 16.92)**
Q4	**5.26 (3.07 – 9.00)**	**13.08 (8.11 – 21.10)**	**6.67 (3.62 – 12.29)**	**12.68 (7.13 – 22.55)**

*Bold values highlight the statistically significant data*.

**Table 5B:** Risk of colorectal cancer according to sex and hemoglobin quartile.

	**Screening group** ***n*** **= 1,515**		**Symptomatic group** ***n*** **= 1,227**	
	**Women**	**Men**	**Women**	**Men**
Q1	1	1.01 (0.22 – 4.56)	1	2.75 (0.82 – 9.16)
Q2	–	1.38 (0.33 – 5.88)	1.31 (0.34 – 5.01)	1.19 (0.31 – 4.51)
Q3	0.46 (0.05 – 4.48)	2.79 (0.77 – 10.09)	2.67 (0.82 – 8.75)	**6.11 (2.04 – 18.32)**
Q4	**5.75 (1.58 – 20.89)**	**5.89 (1.76 – 19.66)**	**12.86 (4.37 – 37.83)**	**16.95 (5.96 – 48.21)**

*Age-adjusted OR (CI95%). Reference Q1 women. Bold values highlight the statistically significant data*.

### Fecal Hemoglobin Concentration According to Colonoscopy Findings Location

FIT concentration was also evaluated according to AN location. Rectal lesions were included in the left-sided group because considering them separately no differences were found. There were statistically significant differences between Hb concentrations in FIT and tumor location, both among the different locations within each group and between groups. FIT values were higher in the symptomatic group compared to the screening group both for left- and right-sided AN. Patients in the screening group that presented left-sided AN had a significantly higher fecal hemoglobin concentration than those with right-sided AN (*p* = 0.034). The risk of AN based on quartiles was always higher for men than for women (data not shown). No differences in hemoglobin values were detected among left- and right-sided lesions in the symptomatic group (Table 6A). When CRC location was evaluated, a similar pattern was observed, but there were no differences among left-sided and right-sided CRC FIT values, neither in symptomatic patients (*p* = 0.426), nor in the screening group (*p* = 0.451) (Table 6B).

**Table 6A:** Hemoglobin FIT values according to colonoscopy findings location (left-sided, right sided).

**Colonoscopy findings**	**Screening group** **(*n* = 1,490) *n* = 1,515**	**Symptomatic group** ***n* = 1,227**	***p*-value^**a**^**
No AN	272.5 (169.0–572.3)	379.0 (189.0–1149.5)	**<0.001**
Left-sided AN	765.0 (306.0–4227.0)	1505.0 (405.0–5983.5)	**0.011**
Right-sided AN	648.0 (242.5–3276.5)	770.0 (329.0–7330.0)	**0.001**
***p*****-value**^**b**^ *overall*	**<0.001**	**<0.001**	
***p*****-value** *no AN* vs*. right-sided*	**<0.001**	**<0.001**	
***p*****-value** *no AN* vs*. left-sided*	**<0.001**	**<0.001**	
***p*****-value** *right-sided* vs*. left-sided*	**0.034**	0.421	

*Bold values highlight the statistically significant data*.

a *Comparison between “SCREENING FIT” and “SYMPTOMATIC FIT” groups*.

b *Comparison between colonoscopy findings within each group*.

**Table 6B:** Hemoglobin FIT values according to colorectal cancer location (left- sided, right-sided).

**Colonoscopy findings**	**Screening group** ***n* = 1,515**	**Symptomatic group** ***n* = 1,227**	***p*-value^**a**^**
No cancer	353.0 (194.0–770.0)	439.0 (211.5–2101.0)	**<0.001**
Left-sided cancer	2852.0 (533.8–8817.0)	5993.0 (768.5–15277.5)	**0.029**
Right-sided cancer	6295.0 (713.0–9694.0)	4683.5 (628.0–12644.3)	0.569
***p*****-value**^**b**^ *overall*	**<0.001**	**<0.001**	
***p*****-value** *no cancer* vs*. right-sided*	**<0.001**	**<0.001**	
***p*****-value** *no cancer* vs*. left-sided*	**<0.001**	**<0.001**	
***p*****-value** *right-sided* vs. *left-sided*	0.451	0.426	

*Median (Q1–Q3). Bold values highlight the statistically significant data*.

a *Comparison between “SCREENING FIT” and “SYMPTOMATIC FIT” groups*.

b *Comparison between colonoscopy findings within each group*.

### Positive Predictive Value (PPV) of FIT for Advanced Neoplasia

**Figure 2:**
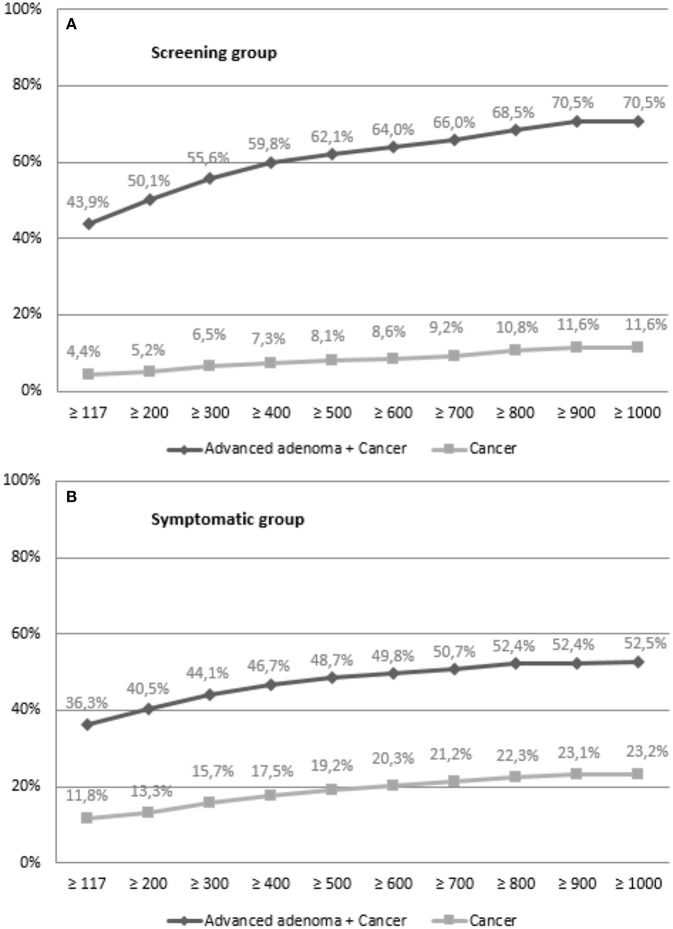
PPV according to fecal hemoglobin concentration in **(A)** screening group and in **(B)** symptomatic group.

Finally, we calculated the PPV of FIT for cancer and advanced adenoma plus cancer (equivalent to advanced neoplasia) using different cut-off values of fecal hemoglobin concentration. As it can be observed in the next figure, PPV increases with higher values of FIT, in each group (Figure 2).

## Discussion

A significant correlation between fecal hemoglobin concentration detected in FIT and the findings of the colonoscopy has been observed in our study, with the amount of hemoglobin detected being higher in the patients with advanced lesions, data consistent with prior studies (13–20). Unlike these studies, here we have shown, in the same study and within the same clinical and laboratory conditions, that these findings can apply not only to the screening group (15–18), but also in patients who referred symptoms (14), which should encourage the use of the FIT in clinical practice as an evaluation of symptomatic patients prior to colonoscopy. This is an important finding since colonoscopy is always planned to be performed below a specified limit of time after testing positive for FIT in screening programs, but this is not the case in patients with symptoms. In this population, FIT still needs to be positioned compared to symptoms in many public, and even private health systems, with waiting lists for colonoscopy which are common due to the growing workload with the implementation of CRC screening programmes and open access to primary care (6, 27–29).

The median fecal hemoglobin value followed an increasing trend according to the severity of the pathology detected in colonoscopy. In cancer, high-risk adenoma, intermediate-risk adenoma and low-risk adenoma the concentration was always higher than in the prior step in both groups, with the only exception of non-neoplastic lesions compared to low-risk adenoma. These results were justifiable, since non-neoplastic lesions included pathologies that might be presented with bleeding, such as inflammatory bowel disease, hemorrhoids or diverticular disease. Other studies have already reported that fecal Hb concentration is related to the presence and severity of lesions, mostly in patients with no symptoms within screening programs (16–18, 23, 30). However, our study provides information for both symptomatic and asymptomatic patients and a more detailed analysis of fecal Hb concentration and risks for each type of neoplastic lesion than that reported in former studies. We show similar risk estimates for AN and cancer in both populations, but symptomatic patients had higher Hb fecal values, which suggests that prediction models based on actual concentration of fecal Hb may need to be different for each population. In our study we cannot provide figures for either specificity or negative predictive values, since our cohorts do not include patients with colonoscopy and negative FIT. Like in our study, Auge et al. (18) analyzed FIT positive patients in one of the Spanish CRC screening programs. They reported similar PPV to those found in our study, although we could show that figures where a bit different between symptomatic (lower values) and asymptomatic patients (higher values) for AN and the opposite for colorectal cancer.

Age and sex have also been proved to be independent risk factors for AN (18). Here, we show a statistically significant difference in the results of colonoscopies according to sex in both groups, and to age in the symptomatic group. These differences were not detected in the asymptomatic population probably due to age limitations in the screening programme in our region. Unlike previous studies (16, 17, 23, 30) we show a more detailed analysis of that risk and provide higher risk values than those reported by Auge et al. (18) in asymptomatic patients. A combination of sex and fecal hemoglobin concentration led to 4 risk categories with different probabilities of presenting an AN, both in screening and symptomatic patients. The patients with the highest risk of presenting AN were male and those with the highest hemoglobin concentration values in the FIT. These findings could be useful to prioritize those individuals with the greatest risk of presenting an AN or cancer in the colonoscopy, especially in centers with large waiting lists.

In this line, several prediction models for symptomatic patients have been developed recently, such as the COLONPREDICT (22), that involved 11 variables (including fecal hemoglobin ≥20 μg/g), and obtained an area under the curve (AUC) = 0.92 (95%CI: 0.91–0.94); or FAST Score (21), a more simple and friendly user model involving FIT hemoglobin concentration, age and sex, with promising results [AUC for CRC detection = 0.88 (CI95%: 0.85–0.90)]. Our results are in line with the FAST score (21) and outline that probably a reliable prediction model with these 3 simple variables (fecal Hb concentration, sex, and age) can be constructed. However, in these studies only the risk of presenting CRC was evaluated. According to our results, fecal hemoglobin concentration could also be used, not only to calculate the risk of CRC, but also AN (CRC plus advanced adenoma). These two scores (21, 22) were validated in symptomatic patients, but similar models with the 3 above mentioned variables could be useful in asymptomatic patients. The evaluation of other variables such as the main symptom, smoking habit, nutritional practice or body mass index, that have not been evaluated in the present study, could also be interesting in the future to continue developing prediction models for advanced neoplasia, but probably the most important and determinant factor will be Hb concentration in FIT. It is possible that adding other measurements such as fecal calprotectin (31) to FIT could improve the diagnostic yield for AN or CRC, but this still need to be proved (32). Risk-stratification models could also be useful to increase the awareness of endoscopists during the procedure about the probability of finding an AN, which could also improve quality indicators such as the adenoma detection rate, strongly correlated with the probability of presenting an interval CRC (33, 34).

On the other hand, it is important to highlight that the risk of presenting advanced neoplasia was similar in the screening group compared to symptomatic patients in the same quartile of hemoglobin concentration detected in the test. Considering colorectal cancer alone both age-adjusted by sex, the risk of presenting CRC was higher in patients who referred symptoms, compared to the asymptomatic ones, but trends showed a similar pattern to those seen for AN. These findings should encourage prioritizing symptomatic patients with a high hemoglobin concentration detected in the FIT.

Unlike other studies (14, 17, 18, 21–23, 30) we report data on FIT values for each type of lesion when colon location is considered. For AN we show higher fecal hemoglobin values in left-sided lesions compared to right-sided in the screening group. However, FIT values were similar in both locations in symptomatic patients, what also occurred with CRC location, in both groups. These data suggest that perhaps for right-sided lesions in screening programs current cut-off values may have different sensitivity and PPVs.

Adjusting the cut-off value of FIT to the available endoscopic resources is an alternative and may be a necessity. Positive predictive value for the different neoplastic lesions was higher when a higher hemoglobin cut-off point was established. Our study showed that a positive FIT (>117 ng/mL) established a 43.9 and 36.3% probability of presenting an advanced adenoma in the screening and symptomatic groups, which raised up to 62.1 and 48.7%, respectively, modifying the cut-off point to 500 ng/mL.

The study has limitations such as the data collection which were collected retrospectively, which limits the main reason and actual symptoms for which the colonoscopy procedure was demanded in the symptomatic group. The construction of appropriate algorithms to automatically classify patients to be prioritized based on the risk of presenting advanced lesions may require that information. In the screening group the age range used is the main limitation, but it was due to the current health policy followed in our regional health system in which the programme was started in patients within this age range as a first step. However, this limitation has made that both populations had a closer age range. We believe that the data agree widely in both populations and these limitations do not invalidate our conclusions.

## Conclusions

The amount of hemoglobin in the fecal immunochemical blood test correlates with the risk of finding neoplastic pathology in the colonoscopy in both asymptomatic and symptomatic patients. Due to the poor sensitivity of symptoms to detect colonic lesions (35), male gender and FIT hemoglobin concentration can be used as predictors of risk of advanced neoplasia and colorectal cancer and to prioritize colonoscopy in patients with positive FIT, both in screening and in symptomatic patients. The need to prioritize patients for colonoscopy is justified based on data that suggest that delays in reaching a CRC diagnosis is associated with worse prognosis, and on the presence of waiting list that can be as long as 6 months (or even longer) in some public universal health systems (28, 29).

## Ethics Statement

This study is retrospective and used data stored in databases, which were anonymized for data analysis. The study was approved by the Regional Ethical Committe of Aragón (CEICA).

## Author Contributions

MN collected data, analyzed data, and drafted the manuscript. GH collected data. TR performed histological analysis. IO collected data and analyzed data. PC-L analyzed data and performed all statistical analysis. AL designed the study, analized data and drafted the manuscript. All authors revised the manuscript and contributed to its intellectual content.

### Conflict of Interest Statement

AL is Advisor to Sysmex Iberia (Barcelona, Spain). The remaining authors declare that the research was conducted in the absence of any commercial or financial relationships that could be construed as a potential conflict of interest.
